# Experimental Inoculation of Egyptian Rousette Bats (*Rousettus aegyptiacus*) with Viruses of the *Ebolavirus* and *Marburgvirus* Genera

**DOI:** 10.3390/v7072779

**Published:** 2015-06-25

**Authors:** Megan E.B. Jones, Amy J. Schuh, Brian R. Amman, Tara K. Sealy, Sherif R. Zaki, Stuart T. Nichol, Jonathan S. Towner

**Affiliations:** 1 Viral Special Pathogens Branch, Centers for Disease Control and Prevention, 1600 Clifton Rd, Atlanta, GA 30333, USA; E-Mails: mjones@sandiegozoo.org (M.E.B.J.); wuc2@cdc.gov (A.J.S.); cxx1@cdc.gov (B.R.A.); tss3@cdc.gov (T.K.S.); stn1@cdc.gov (S.T.N.); 2 Department of Pathology, College of Veterinary Medicine, University of Georgia, 501 D.W. Brooks, Athens, GA 30602, USA; 3 Infectious Diseases Pathology Branch, Centers for Disease Control and Prevention, 1600 Clifton Rd, Atlanta, GA 30333, USA; E-Mail: sxz1@cdc.gov

**Keywords:** *Ebolavirus*, *Marburgvirus*, *Rousettus aegyptiacus*, Egyptian rousette bat, reservoir host, experimental infection study

## Abstract

The Egyptian rousette bat (*Rousettus aegyptiacus*) is a natural reservoir for marburgviruses and a consistent source of virus spillover to humans. Cumulative evidence suggests various bat species may also transmit ebolaviruses. We investigated the susceptibility of Egyptian rousettes to each of the five known ebolaviruses (Sudan, Ebola, Bundibugyo, Taï Forest, and Reston), and compared findings with Marburg virus. In a pilot study, groups of four juvenile bats were inoculated with one of the ebolaviruses or Marburg virus. In ebolavirus groups, viral RNA tissue distribution was limited, and no bat became viremic. Sudan viral RNA was slightly more widespread, spurring a second, 15-day Sudan virus serial euthanasia study. Low levels of Sudan viral RNA disseminated to multiple tissues at early time points, but there was no viremia or shedding. In contrast, Marburg virus RNA was widely disseminated, with viremia, oral and rectal shedding, and antigen in spleen and liver. This is the first experimental infection study comparing tissue tropism, viral shedding, and clinical and pathologic effects of six different filoviruses in the Egyptian rousette, a known marburgvirus reservoir. Our results suggest Egyptian rousettes are unlikely sources for ebolaviruses in nature, and support a possible single filovirus—single reservoir host relationship.

## 1. Introduction

Ebolaviruses and marburgviruses (family *Filoviridae*) are negative-sense, single-stranded RNA viruses that cause severe hemorrhagic fever in humans and non-human primates. Ebola virus disease (EVD) and Marburg virus disease (MVD) are characterized by rapid person-to-person transmission, high case fatality rates, and a lack of approved treatments or vaccines. The family *Filoviridae* is divided into three genera [1]: *Ebolavirus*, *Marburgvirus*, and the genus *Cuevavirus*, recently discovered in a European bat [2]. Genus *Marburgvirus* contains a single species, *Marburg marburgvirus*, with two virus members, Marburg virus (MARV) and Ravn virus (RAVV), which are approximately 20% divergent at the nucleotide level [3]. The genus *Ebolavirus* includes five species, each of which contains a single virus member: *Sudan ebolavirus* (Sudan virus, SUDV), *Zaire ebolavirus* (Ebola virus, EBOV), *Bundibugyo ebolavirus* (Bundibugyo virus, BDBV), *Taï Forest ebolavirus* (Taï Forest virus, TAFV), and *Reston ebolavirus* (Reston virus, RESTV). *Cuevavirus* consists of a single species and virus, *Lloviu cuevavirus* (Lloviu virus). Historically, disease caused by MARV and EBOV have exhibited the highest fatality rates (up to 90% in some outbreaks), followed by SUDV (42%–65%), [4,5,6] and BDBV (36% to 40%) [7,8,9]. TAFV has caused one non-fatal human infection and RESTV is considered non-pathogenic to humans, but both can be highly pathogenic in nonhuman primates.

MVD was first identified in 1967 in Germany and the former Yugoslavia, when laboratory workers acquired a fatal illness after exposure to primates imported from Uganda [10]. EVD was first recorded in 1976 in Zaire (now Democratic Republic of the Congo, DRC) and South Sudan, during concurrent but unrelated outbreaks caused by EBOV and SUDV [11,12]. Since that time, sporadic outbreaks of both MVD and EVD have been recorded, usually involving dozens to hundreds of cases in remote locations in sub-Saharan Africa. The largest ever outbreak of SUDV, and until recently the largest outbreak of any filovirus, occurred in the Gulu district of Uganda in 2000–2001 and involved 425 cases and 224 deaths [13]. The current EBOV outbreak in Western Africa, which surpassed 25,000 cases in March of 2015 [14], represents a significant expansion of case numbers and a new geographic range for the virus, and clearly demonstrates the potential of filoviruses to become significant threats to public health on a global scale. TAFV was discovered in 1994 in Côte d’Ivoire, associated with mortality in wild chimpanzees and one non-fatal human infection [15,16]. RESTV has only been found in the Philippines, or in macaques imported from the Philippines [17,18,19]. Human exposures to RESTV have resulted in seroconversion without clinical signs of disease [20,21]. The most recently discovered ebolavirus, BDBV, was identified in 2007 in Western Uganda, and emerged again in 2012 in DRC [7,9].

The cave-roosting Egyptian rousette bat (*Rousettus aegyptiacus*, also called the Egyptian fruit bat), has been identified as a natural reservoir host for marburgviruses and consistent source of virus spillover to humans [22,23]. This discovery was based on identification of marburgvirus RNA and immunoglobulin G (IgG) [24,25] and the isolation of infectious marburgviruses [22,23,26] from wild rousettes inhabiting caves where human cases had recently occurred. Longitudinal studies have also demonstrated an association between the risk of human infection and the seasonal pulses of active marburgvirus infection in juvenile Egyptian rousettes during biannual reproductive cycles [23]. Cumulative evidence suggests various bat species also play a role in the transmission cycle of ebolaviruses. Epidemiologic links between ebolaviruses and fruit bats were identified in the first SUDV outbreak in 1976 [27] and in TAFV-associated disease in chimpanzees in Côte d’Ivoire in 1994 [15]. Detection of EBOV-specific IgG and, for the first time, RNA was reported in 2005 in fruit bats of three species (*Hypsignathus monstrosus*, *Epomops franqueti*, and *Myonycteris torquata*) hunted for food in Gabon and the Republic of the Congo (RC) [28]. Subsequently, an investigation into a large EBOV outbreak in DRC in 2007 showed a possible link between regional EVD re-emergence and seasonal fruit bat migration [29]. Since that time, several field studies have demonstrated sero-reactivity to EBOV antigen in a variety of fruit bat species, including the Egyptian rousette, in Ghana, Gabon, and RC [28,29,30,31]. Reactivity to recombinant RESTV nucleoprotein were reported in fruit bats in the Philippines [32] and Bangladesh [33], and in eleven different species of insectivorous and fruit bats in China [34]. However, in contrast to results for marburgviruses, repeated attempts at isolation of infectious ebolaviruses from bats have been unsuccessful.

Two recent experimental infection studies of Marburg virus in Egyptian rousettes have demonstrated virus replication in blood and multiple tissues [35,36]; oral shedding of infectious virus [36]; and viral antigen in liver and spleen without evidence of significant disease, findings which are consistent with expectations for a reservoir host. Though numerous field studies have demonstrated potential associations between bats and ebolaviruses, only a single experimental ebolavirus infection study has been conducted in any bat species [37]. In that experiment, a wide range of possible plant, invertebrate, and vertebrate hosts including insectivorous bats of two species (*Mops condylurus*, *Chaerephon pumilus*) and fruit bats of one species (*Epomophorous wahlbergi*) were inoculated with EBOV. Following inoculation, virus was successfully isolated from pooled viscera and blood from bats for up to three weeks, and was isolated from feces in one bat. There was also limited immunohistochemical staining for ebolavirus antigen in pulmonary endothelial cells in one insectivorous bat, without evidence of associated lesions [37]. Recently, a colony of *Mops condylurus* bats was found near the reported index case of the current Western African Ebola virus disease outbreak [38].

EBOV antibodies have been detected in wild Egyptian rousette bats in Gabon [29], and a *R. aegyptiacus*-derived cell line was shown to support EBOV replication *in vitro* [39]. Bats of other *Rousettus* spp. have been seropositive for RESTV and EBOV in the Philippines and China [32,33,34]. However, the capacity for Egyptian rousettes to become infected with ebolaviruses and act as a potential source of infectious virus is not known. Here, we report the findings of an experimental inoculation study of Egyptian rousette bats in which we compare the viral kinetics, tissue and cell tropism, potential for viral shedding, and clinical and pathologic effects of all five known ebolaviruses with findings from Marburg virus. This was a two-part study, consisting of a pilot study to investigate all six filoviruses concurrently, followed by a serial euthanasia study to compare the effects of SUDV infection with our previous findings for MARV. We hypothesized that, if Egyptian rousettes are not a true reservoir host of any of the five species of ebolavirus, then the response of this bat species to experimental infection with ebolaviruses will differ significantly from the response to Marburg virus infection. Inoculation of Egyptian rousette bats with ebolaviruses would result in either (1) abortive infection due to lack of susceptibility; or (2) clinical and pathologic signs of severe disease. We show that Egyptian rousettes are generally refractory to ebolavirus infection and are unlikely to act as sources of infectious virus in nature.

## 2. Materials and Methods

### 2.1. Ethics Statement

All animal procedures and experiments were approved by the Centers for Disease Control and Prevention (CDC) Institutional Animal Care and Use Committee (IACUC) and conducted in strict accordance with the Guide for the Care and Use of Laboratory Animals [40]. The CDC is fully accredited by the Association for Assessment and Accreditation of Laboratory Animal Care International (AAALAC).

### 2.2. Biosafety

All work with infectious virus or infected animals was conducted at the Centers for Disease Control and Prevention (CDC, Atlanta, GA, USA) in a biological safety level-4 (BSL-4) laboratory in accordance with Select Agent regulations (www.selectagents.gov). All investigators and animal care personnel followed international biosafety practices appropriate to BSL-4 and strictly adhered to infection control practices to prevent cross contamination between groups of animals.

### 2.3. Animals and Husbandry

The study animals consisted of juvenile (4–5 months old), first-generation, captive born, Egyptian rousettes (*R. aegyptiacus*) from a marburgvirus- and ebolavirus-free breeding colony founded from wild-caught animals imported from Uganda in 2011 [36]. All husbandry protocols including laboratory caging, diet and feeding schedules, room temperature, humidity, and light cycles were identical to those described in Amman *et al.* [36]. In the BSL-4 laboratory, cages housing each experimental group were maintained in separate isolator units (Duo-Flow Mobile Units, Lab Products Inc., Seaford, DE, USA) to prevent cross-contamination. Bats were group-housed, with a minimum of two, and a maximum of four bats per cage for the pilot study, and a minimum of three and maximum of nine bats per cage for the Sudan virus serial euthanasia study; see below.

### 2.4. Viruses

All virus stocks used in this experiment were titrated using a standard 50% tissue culture infective dose (TCID_50_) protocol on Vero E6 cells and visualized by indirect fluorescent antibody assay (IFA) using appropriate rabbit polyclonal antibodies. For bat inoculations, virus stock was diluted to a concentration of 4 × 10^4^ TCID_50_/mL in sterile Dulbecco’s Modified Eagles Medium (DMEM, Invitrogen, Carlsbad, CA, USA) and each bat received 250 μL of diluted virus, for a dose of 10^4^ TCID_50_ per animal. The strain of Marburg virus used in this and previous experimental infections (371bat virus; see [36]), was originally isolated from a naturally infected Egyptian rousette caught at the Kitaka Mine, Uganda, in 2007 [22] and passaged twice on Vero E6 cells. The ebolavirus stocks used were grown from low-passage seed stocks at the Viral Special Pathogens Branch, Centers for Disease Control and Prevention, as follows: Ebola virus variant Mayinga, originally isolated in 1976 and passaged twice on Vero E6 cells; Sudan virus variant Gulu, originally isolated during the outbreak in Gulu, Uganda in 2000–2001 and passaged three times on Vero E6 cells; Bundibugyo virus originally isolated during the 2007 outbreak in Uganda and passaged twice on Vero E6 cells; Taï forest virus isolated in 1994 and passaged five times on Vero E6 cells, and Reston virus originally isolated from a Rhesus macaque in 1989, and passaged on MA104 cells (×1) and Vero E6 cells (×7). This virus had also been plaque picked and confirmed negative for simian hemorrhagic fever virus.

### 2.5. Ebolavirus Pilot Study

This was a 10-day pilot study to investigate the response of Egyptian rousettes to experimental infection of each of the five ebolavirus species. Four bats (2 male and 2 female) were randomly assigned to each experimental group, to be inoculated with either MARV, EBOV, SUDV, BDBV, TAFV, or RESTV; two bats (1 male and 1 female) were randomly assigned as mock-inoculated controls. Experimental inoculation procedures were performed as in in Amman *et al.* [36]. Briefly, bats were lightly anesthetized using isoflurane anesthetic and inoculated subcutaneously in the ventral abdomen with 250 μL of virus stock diluted in DMEM, for a total dose of 10^4^ TCID_50_ of virus per animal. Control animals were inoculated with 250 μL of DMEM only. Two animals (one male, one female) from each group were scheduled for euthanasia at 5 and 10 days post-inoculation (DPI), and both mock-inoculated animals were euthanized on day 10. Body weights, rectal temperatures, and blood samples for Q-RT-PCR and complete blood counts (CBC) were obtained prior to infection and then daily from 1 DPI until the time of euthanasia. Due to a larger volume requirement (100 μL) and blood sampling limits for this species, sufficient blood for chemistry analysis was only available on the day of euthanasia. Oral and rectal swab samples were taken daily as described in Amman *et al*. [36]. Animals were euthanized under deep isoflurane anesthesia by exsanguination via cardiac puncture.

### 2.6. Sudan Virus (Variant Gulu) Serial Euthanasia Study

This was a 15-day serial euthanasia study to investigate viral infection kinetics, tissue and cell tropism, potential for viral shedding, and clinical and pathologic findings, of Egyptian rousette bats inoculated with SUDV (variant Gulu). This study was designed to complement our previous Marburg virus serial euthanasia study [36], while taking into account the limited number of juvenile, single-cohort bats available from the breeding colony at one time. Sample collection time points were chosen for direct comparison with days 3, 6, 9, and 12 of the MARV study, and an additional time point was added at 15 DPI. Twenty-one juvenile (4–5 month old) Egyptian rousettes were randomly assigned to be inoculated with 10^4^ TCID_50_ of Sudan virus (*n* = 15 bats), 10^4^ TCID_50_ of Marburg virus (*n* = 3), or mock inoculated (*n* = 3). Viruses, inoculation procedures, dosages, and volumes were identical to those in the pilot study, above. Rectal temperatures, oral swabs, and blood samples for Q-RT-PCR and CBC were obtained prior to infection and then daily starting at 1 DPI until euthanasia, as described above. Body weights were obtained prior to infection and then on days 3, 6, 9, 12, and 15. Three SUDV-inoculated bats (2 males, 1 female; sex ratios were determined by available animals of appropriate age in our breeding colony) were scheduled for euthanasia on each of 3, 6, 9, 12, and 15 DPI, and euthanasia procedures were as described above. MARV-inoculated and mock-inoculated bats were euthanized at 15 DPI. Blood was sampled for chemistry analysis from each bat on the day of euthanasia. Blood was taken for serology at 0, 5, 10, and 15 DPI.

### 2.7. Hematology and Clinical Chemistry

For daily CBCs, blood was collected from the cephalic vein into a 20 μL, EDTA-coated capillary tube (True20 capillary tube) and analyzed using a Hematrue blood analyzer (HESKA, Loveland, CO, USA). For blood chemistry profiles, 100 μL of lithium heparinized whole blood were analyzed using Comprehensive Metabolic Panel Discs for the Piccolo point of care chemistry analyzer (Abaxis, Union City, CA, USA). Chemistry analyses included alanine aminotransferase (ALT), albumin, alkaline phosphatase (ALP), aspartate aminotransferase (AST), calcium, chloride, creatinine, glucose, potassium, sodium, total bilirubin, total carbon dioxide, total protein, and blood urea nitrogen (BUN). CBC and chemistry values were compared to reference ranges generated from samples collected and analyzed using identical protocols, from healthy juvenile Egyptian rousette bats in our colony.

### 2.8. Necropsy

Complete necropsies were performed immediately following euthanasia. For RNA extraction, approximately 100 mg samples of each tissue were collected with sterile instruments to prevent cross-contamination. For the ebolavirus pilot study, this included liver, spleen, skin from the inoculation site, skin from the antebrachium, axillary lymph node, lung, heart, kidney, adrenal gland, small intestine, large intestine, mesenteric lymph node, gonad, urinary bladder, and salivary gland. For the Sudan virus serial euthanasia study, tissues collected for RNA extraction were liver, spleen, skin from the inoculation site, axillary lymph node, lung, heart, kidney, small intestine, large intestine, gonad, urinary bladder, and salivary gland. Tissue samples collected for histologic examination were fixed by immersion in 10% neutral buffered formalin in the BSL-4 laboratory for a minimum of 7 days, and then formalin was completely replaced prior to further processing. Tissues collected and processed for histopathology for both the pilot study and the serial euthanasia study included liver, spleen, lung, heart, trachea, thymus, tongue, tonsils, stomach, small intestine, pancreas, large intestine, mediastinal lymph nodes, kidney, adrenal gland, salivary gland, mandibular lymph node, axillary lymph node, pectoral muscle, skin from inoculation site, skin from antebrachium, and skin from patagium (wing membrane).

### 2.9. RNA Extraction and Q-RT-PCR

RNA extraction for blood, tissues, oral swabs, and rectal swabs was performed as described in Amman *et al.* [36]. Quantitative reverse-transcriptase PCR (Q-RT-PCR) was performed using the SuperScript III Platinum One-Step qRT-PCR kit, Invitrogen/Life Technologies) and routine diagnostic protocols targeting Marburg virus VP40, NP of Ebola virus, Sudan virus, and Reston virus, and the VP40 of Bundibugyo and Taï Forest viruses. Standard curves for Q-RT-PCR results for the Sudan virus serial euthanasia study were generated from ten-fold serial dilutions of the Marburg and Sudan virus stocks used in infections, and added to blood, tissue (calf liver) homogenate, or DMEM in the same proportions as experimental blood, tissue, or swab samples, respectively. The relative TCID_50_ equivalent per mL (fluids) or g (tissue) for experimental samples were interpolated from the relevant standard curve.

### 2.10. Histology and Immunohistochemistry

Representative sections of all formalin-fixed tissues were embedded in paraffin, sectioned at 4 micrometers, mounted on glass slides, and routinely stained with hematoxylin and eosin (HE) for histologic examination.

Immunohistochemical staining was performed using an alkaline-phosphatase (AP) polymer detection system (UltraVision Detection System, Thermo Scientific, Waltham, MA, USA). Four-micron sections of formalin-fixed, paraffin-embedded tissues were deparaffinized and rehydrated, then subjected to proteinase-K (Roche, Pleasanton, CA, USA) digestion for 15 min at room temperature (RT). Ultra V Block (Thermo Scientific) was applied for 10 min at RT. The primary antibody was either a rabbit anti-Marburg virus polyclonal or a rabbit anti-ebolavirus polyclonal antibody (Viral Special Pathogens Branch, Centers for Disease Control and Prevention, Atlanta, GA, USA), diluted to 1:250 and incubated for 30 min at RT, followed by Primary Antibody Enhancer (Thermo Scientific; 10 min at RT). AP Polymer (Thermo Scientific) was used as the secondary antibody at manufacturer’s dilution and incubated for 15 min at RT. The detector was Naphthol Phosphate Substrate/Fast Red (Thermo Scientific; 20 min at RT). Sections were counterstained with Mayer’s modified hematoxylin (Poly Scientific, Bay Shore, NY, USA). For negative controls, replicate sections from each block were deparaffinized and stained in parallel following an identical protocol, with the primary antibody replaced by normal rabbit serum (Centers for Disease Control and Prevention, Atlanta, GA, USA).

### 2.11. Serology

In the SUDV serial euthanasia study, blood samples taken for serologic analysis were tested by ELISA for the presence of IgG antibodies reactive to SUDV, as described in Ksiazek *et al.* [41,42] with the modification that 96-well plates were coated with 50 ng/well of recombinant SUDV (variant Gulu) nucleocapsid protein (NP) expressed in *E. coli* and sum ODs adjusted by subtracting reactivity at each 4-fold dilution (1:100 to 1:6400) to Lassa virus (strain Josiah) NP similarly expressed and purified from *E. coli*.

### 2.12. Statistical Analyses

Statistical analyses were performed using Prism 6.0 (GraphPad Software, La Jolla, CA, USA) and Stata 13 (StataCorp, College Station, TX, USA). For each blood chemistry parameter, values from infected animals at each time point (*n* = 3 per time point) were compared with those of mock-inoculated bats (*n* = 3) using one-way analysis of variance (ANOVA), followed by Dunnet’s multiple comparison test if the ANOVA demonstrated significant differences between groups (*p <* 0.05). Data obtained at multiple time points for each individual bat (CBC and weight data) were analyzed using repeated measures ANOVA.

## 3. Results

### 3.1. Ebolavirus Pilot Study

#### 3.1.1. Clinical and Hematologic Findings

**Figure 1:**
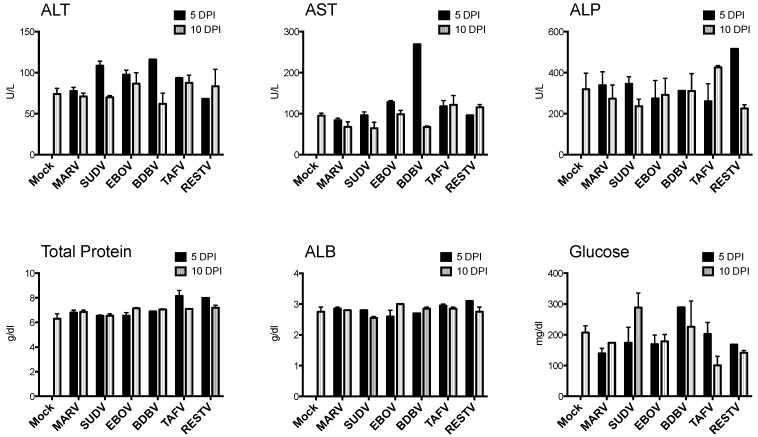

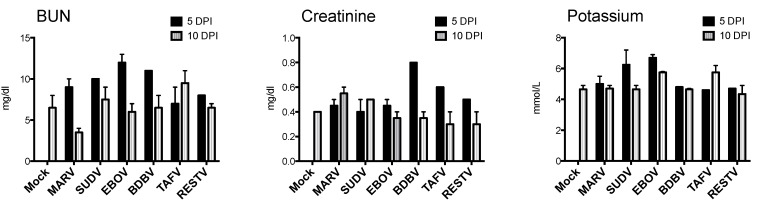
Blood chemistry measurements for bats inoculated with six different filoviruses in the pilot study and euthanized at 5 and 10 days post inoculation (DPI). Mock = mock-inoculated controls, MARV = Marburg virus, SUDV = Sudan virus, EBOV = Ebola virus, BDBV = Bundibugyo virus, TAFV = Taï Forest virus, and RESTV = Reston virus. ALT = alanine aminotransferase, AST = aspartate aminotransferase, ALP = alkaline phosphatase, ALB = albumin, BUN = blood urea nitrogen.

No clinical signs or behavioral changes suggestive of morbidity were observed in any animal, and there were no mortalities. Bats included in the pilot study weighed 109.9 g ± 11.5 (mean ± SD), with a range of 80.8 g to 129.1 g, and there was no mean weight difference between experimental groups (F_6,19_ = 2.491, *p* > 0.05). Change in percent daily body weight did not significantly differ between groups. Over the course of the experiment, most bats tended to gain weight, with a maximum gain of 10.1% over 10 days, and no individual animal lost more than 2% body weight, relative to initial weight. Rectal temperatures remained within normal ranges in all animals. Blood chemistry data for the pilot study are shown in Figure 1. AST was elevated (269 U/L; normal range 26–136 for juvenile bats in our colony) in one BDBV-inoculated bat at 5 DPI. Other parameters remained within normal limits for all bats.

**Figure 2:**
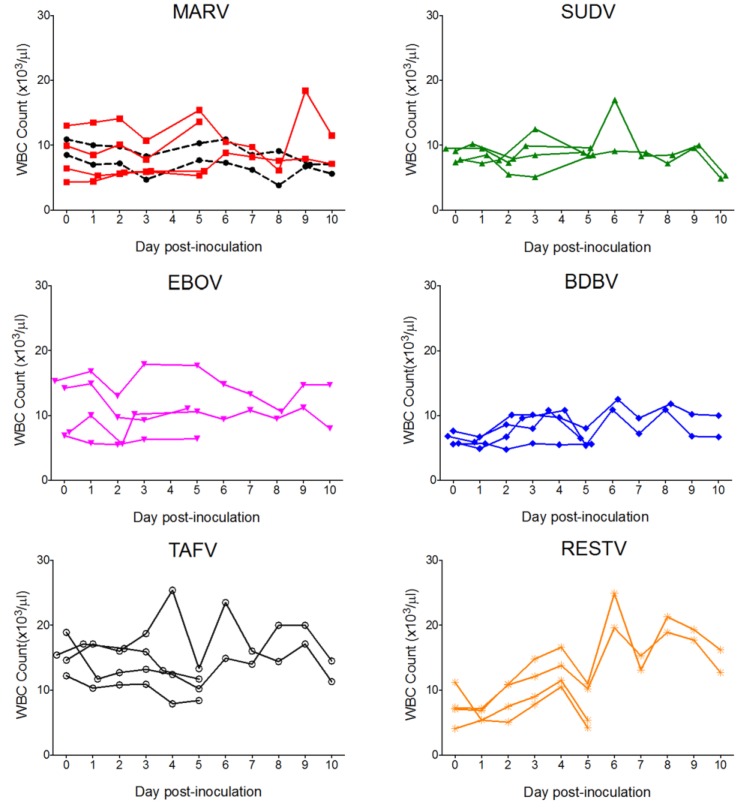
Complete white blood cell (WBC) counts for bats inoculated with six different filoviruses in the pilot study and euthanized at 5 and 10 days post inoculation (DPI). Mock = mock-inoculated controls, MARV = Marburg virus, SUDV = Sudan virus, EBOV = Ebola virus, BDBV = Bundibugyo virus, TAFV = Taï Forest virus, and RESTV = Reston virus.

CBC data are shown in Figure 2. Overall, WBC counts for EBOV, TAFV, and RESTV bats were higher than those for controls, MARV, or SUDV, though all WBC parameters remained within the normal range for all but two bats. One TAVF-inoculated bat and one RESTV bat exhibited mild leukocytosis characterized by monocytosis and lymphocytosis on days 4 and 6 and days 6 and 8 post-inoculation, respectively. Platelet and erythrocyte counts remained within normal limits for all bats.

#### 3.1.2. Q-RT-PCR

**Figure 3:**
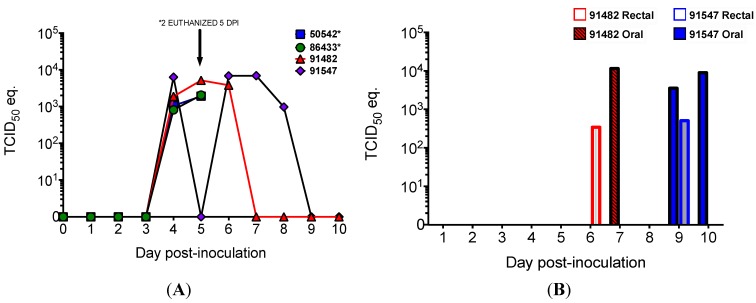
Viral loads, as determined by Q-RT-PCR and expressed as 50% tissue culture infective dose (TCID_50_) equivalents per mL, in four bats inoculated with Marburg virus in the Pilot Study and euthanized at 5 (*n* = 2) and 10 (*n* = 2) DPI. (**A**) Marburg viral RNA in blood is evidence of viremia in all four Marburg virus-inoculated bats; (**B**) Marburg viral RNA in oral (filled bars) and rectal (open bars) swabs.

Viral RNA was never detected in the blood of any of the ebolavirus-inoculated or mock-inoculated bats. All four MARV-inoculated bats became viremic (as determined by the presence of viral RNA in blood) at 4 DPI, and MARV RNA was detected for at least two days in each bat (Figure 3). Both bats euthanized at 5 DPI were viremic at the time of euthanasia, and viremia was detected until days 7 and 8 in the two MARV bats euthanized on day 10.

The viral tissue distribution and levels of viral RNA for the pilot study are summarized in Table 1. MARV was widely disseminated in bats euthanized at 5 and 10 DPI, with RNA detected in a total 11 of 16 tissue types tested. RNA was most frequently detected in skin at the inoculation site (*n* = 4), liver (*n* = 4), spleen (*n* = 3), and salivary gland (*n* = 3), but was also found in axillary lymph node (*n* = 1), urinary bladder (*n* = 2), small intestine (*n* = 2), mesenteric lymph node (*n* = 1), gonad (*n* = 2, both males), heart (*n* = 1), and kidney (*n* = 1). SUDV RNA was detected in a total of five of 16 different tissue types tested from four bats (Table 1), including skin from the inoculation site (*n* = 3), liver (*n* = 2), spleen (*n* = 2), axillary lymph node (*n* = 3), and urinary bladder (*n* = 1). For EBOV, BDBV, and RESTV, RNA dissemination was limited to skin from the inoculation site and axillary lymph node, and for TAFV, only the inoculation site was PCR-positive (Table 1). All oral and rectal swabs from all five ebolavirus groups and mock-inoculated bats were negative by Q-RT-PCR. In contrast, MARV RNA was detected in oral and rectal swabs from both MARV-inoculated bats euthanized at 10 DPI (Figure 3).

**Table 1:** Pilot Study. Tissue viral loads as determined by quantitative reverse-transcriptase PCR (Q-RT-PCR) ^a,b,c^ for Egyptian rousette bats (*Rousettus aegyptiacus*) experimentally inoculated with Marburg virus or one of five ebolaviruses, and euthanized at days 5 or 10 post-inoculation. Tissues in which viral antigen was detected by immunohistochemistry are marked with an asterisk (*).

Virus	DPI	Bat ID	Sex	Skin (Inoc)	Liv	Spl	Ax LN	Saliv G	UrBl	S Int	Mes LN	G	Hrt	Kid	Bld
Mock	10	85334	f	**−**	**−**	**−**	**−**	**−**	**−**	**−**	**−**	**−**	**−**	**−**	**−**
		91271	m	**−**	**−**	**−**	**−**	**−**	**−**	**−**	**−**	**−**	**−**	**−**	**−**
MARV	5	86433	f	**++++** *	**+++** *	**+++** *	**++**	**++**	**++**	**−**	**−**	**−**	**−**	**−**	**++**
		50542	m	**++++** *	**++++** *	**+++** *	**−**	**−**	**++**	**−**	**−**	**++**	**++**	**−**	**++**
	10	91482	f	**++++** *	**++**	**−**	**−**	**++**	**−**	**++**	**−**	**−**	**−**	**+++**	**-**
		91547	m	**+++** *	**+++**	**+++**	**−**	**+++**	**−**	**++**	**++**	**++**	**−**	**−**	**−**
SUDV	5	56380	f	**++** *	**+**	**+**	**+**	**−**	**−**	**−**	**−**	**−**	**−**	**−**	**−**
		16107	m	**++**	**+**	**+**	**−**	**−**	**−**	**−**	**−**	**−**	**−**	**−**	**−**
	10	43612	f	**+**	**−**	**−**	**+**	**−**	**+**	**−**	**−**	**−**	**−**	**−**	**−**
		20778	m	**−**	**−**	**−**	**+**	**−**	**−**	**−**	**−**	**−**	**−**	**−**	**−**
EBOV	5	85933	f	**++** *	**−**	**−**	**−**	**−**	**−**	**−**	**−**	**−**	**−**	**−**	**−**
		52392	m	**−**	**−**	**−**	**−**	**−**	**−**	**−**	**−**	**−**	**−**	**−**	**−**
	10	41902	f	**++**	**−**	**−**	**++**	**−**	**−**	**−**	**−**	**−**	**−**	**−**	**−**
		26060	m	**+**	**−**	**−**	**−**	**−**	**−**	**−**	**−**	**−**	**−**	**−**	**−**
BDBV	5	41354	f	**++**	**−**	**−**	**−**	**−**	**−**	**−**	**−**	**−**	**−**	**−**	**−**
		91128	m	**++**	**−**	**−**	**−**	**−**	**−**	**−**	**−**	**−**	**−**	**−**	**−**
	10	23796	f	**−**	**−**	**−**	**−**	**−**	**−**	**−**	**−**	**−**	**−**	**−**	**−**
		25844	m	**−**	**−**	**−**	**++**	**−**	**−**	**−**	**−**	**−**	**−**	**−**	**−**
TAFV	5	42084	f	**++**	**−**	**−**	**−**	**−**	**−**	**−**	**−**	**−**	**−**	**−**	**−**
		35825	m	**+++**	**−**	**−**	**−**	**−**	**−**	**−**	**−**	**−**	**−**	**−**	**−**
	10	42348	f	**−**	**−**	**−**	**−**	**−**	**−**	**−**	**−**	**−**	**−**	**−**	**−**
		26015	m	**+**	**−**	**−**	**−**	**−**	**−**	**−**	**−**	**−**	**−**	**−**	**−**
RESTV	5	86551	f	**+++** *	**−**	**−**	**−**	**−**	**−**	**−**	**−**	**−**	**−**	**−**	**−**
		38558	m	**++**	**−**	**−**	**−**	**−**	**−**	**−**	**−**	**−**	**−**	**−**	**−**
	10	50188	f	**++**	**−**	**−**	**−**	**−**	**−**	**−**	**−**	**−**	**−**	**−**	**−**
		45164	m	**−**	**−**	**−**	**++**	**−**	**−**	**−**	**−**	**−**	**−**	**−**	**−**

^a^ Abbreviations for tissues: Skin (Inoc) = skin taken from inoculation site; Liv = liver, Spl = spleen, Ax LN = axillary lymph node, Saliv G = salivary gland, UrBl = urinary bladder, S Int = small intestine, Mes LN = mesenteric lymph node, G = gonad, Hrt = heart, Kid = kidney, and Bld = blood at time of euthanasia. Abbreviations for viruses: Mock = mock inoculated (control), MARV = Marburg virus, SUDV = Sudan Virus, EBOV = Ebola virus, BDBV = Bundibugyo virus, TAFV = Taï Forest virus, RESTV = Reston virus. DPI = day post-inoculation; ^b^ Tissue viral load as indicated by cycle threshold (*C*t) value from Q-RT-PCR assay: + = *C*t 35–40, ++ = *C*t 30–34.9, +++ *C*t 25–29.9, ++++ *C*t 20–24.9; ^c^ Tissues also tested that were negative for all animals included adrenal gland, lung, large intestine, brain, and skin from antebrachium.

#### 3.1.3. Necropsy, Histopathology, and Immunohistochemistry

**Figure 4:**
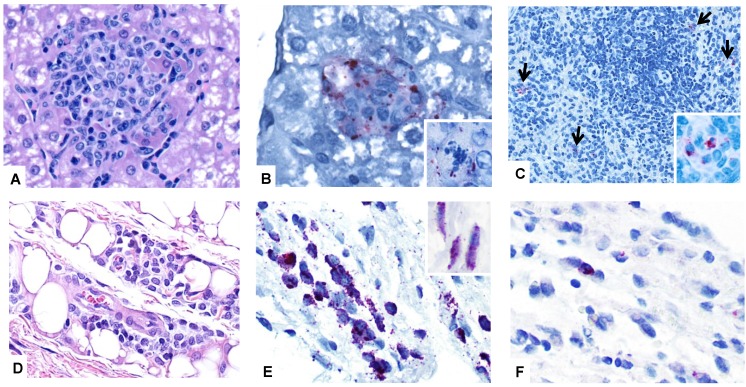
Photomicrographs of tissues from Egyptian rousette bats experimentally inoculated with filoviruses. (**A**) Liver, MARV-inoculated bat, day 5 post-inoculation (pilot study). A focus of histiocytic infiltrate and rare necrotic hepatocytes disrupts the parenchyma. H&E stain; (**B**) Liver, MARV-inoculated bat, day 5 post-inoculation (pilot study). Marburgviral antigen (red) is present in the cytoplasm of hepatocytes and macrophages in a small focus of infiltrate. Inset: positive immunostaining in the cytoplasm of a necrotic hepatocyte from the same section. Immunoalkaline phosphatase with naphthol fast red and hematoxylin counterstain; (**C**) Spleen, MARV-inoculated bat, 5 days post-inoculation (pilot study). MARV antigen is present in small numbers of red pulp macrophages (arrows). Inset: Higher magnification of cytoplasmic antigen in macrophages. Immunoalkaline phosphatase with naphthol fast red and hematoxylin counterstain; (**D**) Skin (subcutaneous tissue) from the inoculation site, MARV-inoculated bat, 5 days post-inoculation (pilot study). A small focus of macrophages infiltrates the subcutis at the site of MARV inoculation. H&E stain; (**E**) Skin (subcutaneous tissue) from the inoculation site, MARV-inoculated bat, 5 days post-inoculation (pilot study). Positive immunostaining in subcutaneous macrophages (main panel) and fibroblasts (inset) at the inoculation site. Immunoalkaline phosphatase with naphthol fast red and hematoxylin counterstain; (**F**) Skin (subcutaneous tissue) from the inoculation site, SUDV-inoculated bat, 3 days post-inoculation (SUDV serial euthanasia study). Cytoplasmic antigen (red) in macrophages at the inoculation site. Immunoalkaline phosphatase with naphthol fast red and hematoxylin counterstain.

Necropsy revealed no significant gross lesions in any bat from any experimental group. All animals had abundant abdominal and subcutaneous adipose tissue. On histologic examination of the liver, most bats exhibited moderate to marked, midzonal to diffuse hepatocellular vacuolation, consistent with glycogen accumulation, a common incidental finding in in our colony. The distribution and degree of vacuolation was similar in all experimental groups including controls. In the livers of MARV-inoculated bats, there were small, randomly scattered aggregates of cellular infiltrate composed predominantly of histiocytes and lymphocytes admixed with few neutrophils (Figure 4). These foci sometimes contained necrotic or apoptotic hepatocytes and karyorrhectic debris. Foci were most frequent in animals with higher viral load in the liver. IHC staining for MARV in the liver revealed antigen in a small proportion of these foci in both bats from 5 DPI (Figure 4; Table 1). Positive, cytoplasmic, granular to globular immunostaining was localized to histiocytes or hepatocytes, and was sometimes perimembranous in hepatocytes. Very rarely, small foci of similar liver infiltrate were also present in one SUDV bat (10 DPI), one EBOV bat (10 DPI), one BDBV bat (10 DPI), two TAFV bats (both from 10 DPI), two RESTV bats (5 and 10 DPI), and one control bat (10 DPI), but immunohistochemical stains of liver were negative for all ebolavirus-inoculated and mock-inoculated bats.

In the spleen, small amounts of MARV antigen were present in the cytoplasm of red pulp histiocytes in both bats from 5 DPI (Figure 4). No splenic lesions were identified in any bat, and no ebolavirus antigen was detected in spleen in any ebolavirus-inoculated or mock-inoculated bat.

In all experimental groups, histologic examination of skin from the inoculation site revealed small aggregates of macrophages in the deep subcutaneous tissues that decreased in cell density from 5 to 10 DPI. These aggregates were present in all MARV- and all SUDV-inoculated bats, but were larger in MARV bats than in other groups. In other virus-inoculated groups, only three of four bats had comparable lesions. Immunohistochemical staining of inoculation site skin sections demonstrated MARV antigen in the cytoplasm of subcutaneous histiocytes and fibroblast-type in all four MARV-inoculated bats, though antigen was sparse at 10 DPI (Figure 4). Very small amounts of virus-specific antigen was also present in histiocytes and, rarely, fibroblasts at 5 DPI in bats inoculated with SUDV (*n* = 1), EBOV (*n* = 2), and RESTV (*n* = 1). All other tissues examined by immunohistochemical staining were negative in all bats.

### 3.2. Sudan Virus Serial Euthanasia Study

Based on pilot study Q-RT-PCR results, which showed SUDV to be more widely disseminated than the other ebolavirus species, SUDV was further investigated in a serial euthanasia study. This study was designed to complement our previous Marburg virus serial euthanasia study [36], while also taking into account the limited number of juvenile, single-cohort bats available from the breeding colony at one time. Euthanasia and other sampling time points were chosen for direct comparison with days 3, 6, 9, and 12 of the MARV study, and an additional time point was added at 15 DPI.

#### 3.2.1. Clinical and Hematologic Findings

**Figure 5:**
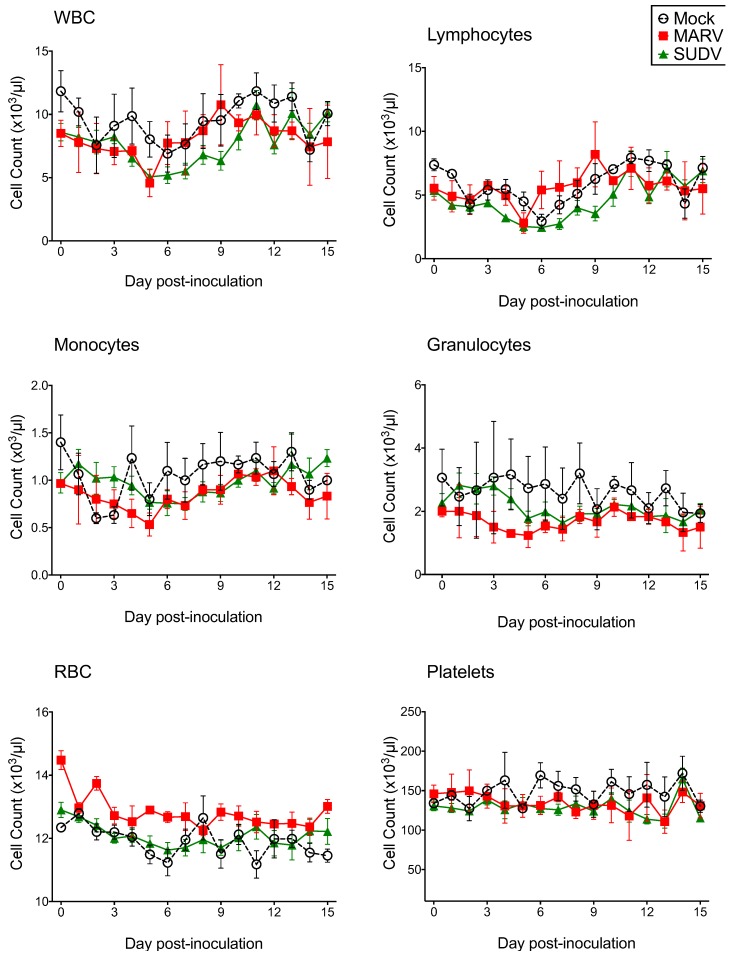
Complete blood count data for Egyptian rousette bats inoculated with Sudan virus (*n* = 15, green triangles), Marburg virus (*n* = 3, red squares) and mock-inoculated controls (*n* = 3, open circles/dashed line) in a serial euthanasia study. WBC = white blood cell count, RBC = red blood cell count, MARV = Marburg virus, SUDV = Sudan virus.

**Figure 6:**
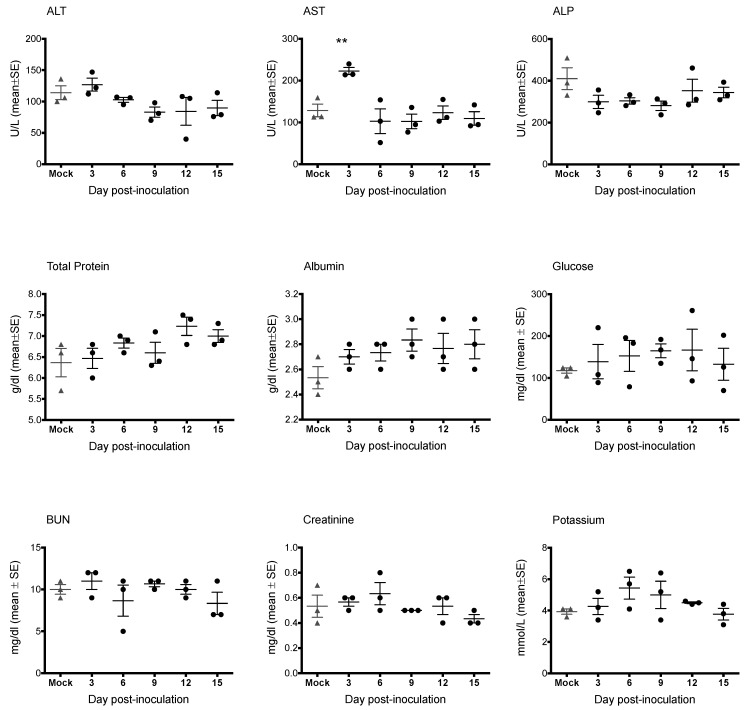
Blood chemistry measurements for Egyptian rousette bats inoculated with Sudan virus. Three Sudan virus-inoculated bats were euthanized on each of days 3, 6, 9, 12 and 15 post-inoculation. Mock-inoculated bats were euthanized on day 15. Mock = mock-inoculated controls, SUDV = Sudan virus, ALT = alanine aminotransferase, AST = aspartate aminotransferase, ALP = alkaline phosphatase, ALB = albumin, BUN = blood urea nitrogen.

As in the pilot study, there were no mortalities and no evidence of significant clinical disease. Bats included in the study weighed 99.0 ± 12.2 g (mean ± SD), with a range of 72.0 to 120.6 g, and there was no significant weight difference between groups (F_2,18_ = 0.29; *p* = 0.750). Percent weight change per time point (every 3 days) did not significantly differ between groups, and average weights for each group tended to increase over time. CBC results are shown in Figure 5. CBC parameters remained within the normal range for all bats. Relative to day 0, average counts of total white blood cells, lymphocytes, and monocytes for all groups tended to decrease until approximately 4–5 DPI, then increase to peak at day 9–11. Granulocytes, platelets, and erythrocyte counts remained relatively stable from day to day. There were no statistical differences in any CBC parameter between virus groups. Blood chemistry results are shown in Figure 6. AST was significantly elevated in SUDV bats at 3 DPI relative to all other days (F_6,14_ = 6.411, *p* = 0.002). No other chemistry value was significantly elevated.

#### 3.2.2. Q-RT-PCR

RNA was never detected in the blood of any SUDV-inoculated bat. All 3 MARV bats became viremic (as indicated by detection of MARV RNA in blood) at 5 DPI and remained so for 2 (*n* = 2) to 3 (*n* = 1) days. Q-RT-PCR results for tissues for SUDV bats from days 3–15 are shown in Table 2. SUDV RNA was most frequently detected in the skin from the inoculation site (*n* = 13), axillary lymph node (*n* = 7), large intestine (*n* = 5), and urinary bladder (*n* = 4). Liver was PCR positive in 4 bats, at 3, 6, and 15 DPI, at lower viral loads than in MARV-inoculated bats in our previous study [36]. SUDV RNA was detected in spleen in three bats total, two at 3 DPI and one at 6 DPI. Tissue viral loads were greatest in skin from the inoculation site and in spleen, and in both sites were detected at levels greater than the inoculation dose 10^4^ TCID_50_/g equivalent, consistent with viral replication. Other PCR-positive tissues included small intestine (*n* = 2), gonad (*n* = 3), heart (*n* = 1) and kidney (*n* = 3). SUDV RNA was never detected in salivary gland or oral or rectal swabs.

**Table 2:** Tissue viral loads ^a^ for Egyptian rousette bats (*Rousettus aegyptiacus*) inoculated with Sudan virus (Gulu) in a serial euthanasia study. Tissues in which Sudan virus antigen was identified are marked with an asterisk (*) ^b,c^.

Group	DPI	Bat ID	Skin (Inoc)	Liv	Spl	Ax LN	Ur Bl	S Int	Lg Int	G	Hrt	Kid
SUDV	3	546948	**++++** *	**−**	**+**	**++**	**+**	**+**	**++**	**++**	**−**	**−**
		684640	**+++++** *	**−**	**−**	**+++**	**+**	**−**	**+**	**+**	**−**	**−**
		720747	**++++** *	**++**	**++++**	**+++** *	**−**	**−**	**+**	**+**	**−**	**−**
	6	550595	**+++**	**−**	**+**	**+++**	**−**	**−**	**−**	**−**	**−**	**−**
		556705	**++++**	**−**	**−**	**++**	**+**	**+**	**++**	**−**	**−**	**−**
		690641	**++++** *	**+**	**−**	**−**	**++**	**-**	**+**	**−**	**−**	**−**
	9	725908	**+++**	**−**	**−**	**−**	**−**	**−**	**−**	**−**	**−**	**−**
		845660	**++**	**−**	**−**	**−**	**−**	**−**	**−**	**−**	**−**	**−**
		546543	**+**	**−**	**−**	**−**	**−**	**−**	**−**	**−**	**−**	**−**
	12	721126	**++**	**−**	**−**	**+++**	**−**	**−**	**−**	**−**	**−**	**−**
		724099	**+**	**−**	**−**	**−**	**−**	**−**	**−**	**−**	**−**	**−**
		684978	**−**	**−**	**−**	**+**	**−**	**−**	**−**	**−**	**−**	**−**
	15	642832	**++**	**−**	**−**	**−**	**−**	**−**	**−**	**−**	**−**	**−**
		721018	**++**	**−**	**−**	**−**	**−**	**−**	**−**	**−**	**−**	**−**
		723995	**−**	**+**	**−**	**−**	**−**	**−**	**−**	**−**	**−**	**−**
Mock	15	214528	**−**	**−**	**−**	**−**	**−**	**−**	**−**	**−**	**−**	**−**
		550277	**−**	**−**	**−**	**−**	**−**	**−**	**−**	**−**	**−**	**−**
		684727	**−**	**−**	**−**	**−**	**−**	**−**	**−**	**−**	**−**	**−**

^a^ Viral loads are expressed as 50% tissue culture infective dose (TCID_50_) equivalents per gram, derived from standard curves of the diluted stock viruses assayed using the identical Q-RT-PCR protocols as that for tissues: **+** <100 TCID_50_/g eq.; **++** 100–999 TCID_50_/g eq.; **+++** 1000–9999 TCID_50_/g eq.; **++++** 10,000–100,000 TCID_50_/g eq; ^b^ Abbreviations: Skin (Inoc) = skin from the inoculation site (ventral abdomen); Liv = liver; Spl = spleen; Ax LN = axillary lymph node; Ur Bl = urinary bladder; S Int = small intestine; G = gonad; Hrt = heart; Kid = kidney; SUDV = Sudan virus; ^c^ Tissues also tested that were negative in all animals: Lung, salivary gland.

#### 3.2.3. Necropsy, Histology, and Immunohistochemistry

Histologic findings from SUDV bats were comparable to those in the pilot study. At 3 DPI, one animal had very few, randomly scattered foci of mononuclear infiltrate in the liver, and similar foci were present in all three bats at day 6. These foci were still present in on days 9 (*n* = 2) and 12 (*n* = 1), and sometimes contained single to few necrotic hepatocytes. Also similar to the pilot study, there were small, subcutaneous aggregates of macrophages in deep adipose tissue at the inoculation site (Figure 4). SUDV antigen was only detected in tissues with higher viral loads (Table 2): Antigen was present in very small numbers of macrophages in the deep subcutis of the inoculation site in 4 bats from 3 and 6 DPI, and one bat had a small amount of SUDV antigen in an axillary lymph node. No MARV-antigen was detected at 15 DPI, and all control bats were negative.

#### 3.2.4. Serology

**Figure 7:**
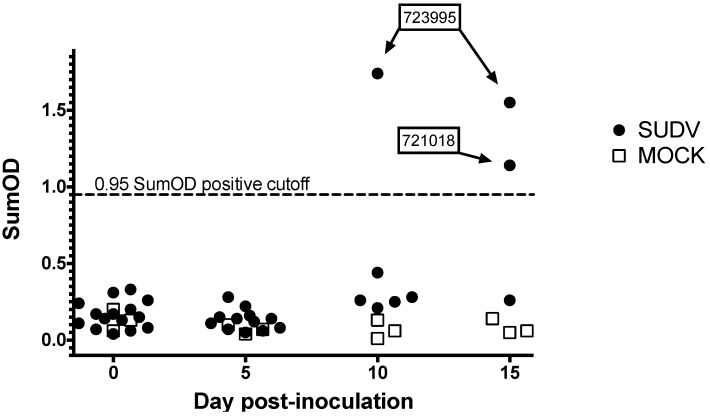
Serology results for Egyptian rousette bats inoculated with Sudan virus in a serial euthanasia study. Results for anti-SUDV IgG measured by enzyme linked immunosorbent assay are shown as adjusted sum optical densities (OD) by day post-inoculation for 15 SUDV inoculated bats (black circles) and 3 mock-inoculated control bats (open squares). Numeric labels represent individual animal identification numbers for two bats that seroconverted.

Serology results are shown in Figure 7. One of six bats remaining at 12 DPI had seroconverted, and a second bat seroconverted on day 15. IgG was not detected in mock inoculated control bats.

## 4. Discussion

This is the first reported experimental infection study comparing the viral kinetics, tissue and cell tropism, and clinical and pathologic effects across six different filovirus species, in a bat host known to act as a natural reservoir for Marburg virus. The pilot study, in which four animals each were inoculated with identical doses of SUDV, EBOV, BDBV, TAFV, RESTV, and MARV, showed that tissue dissemination of ebolaviruses was limited in Egyptian rousettes, viremia was not detected, and there was no evidence of viral shedding via oral or fecal routes. In contrast, Marburg virus was detected in the blood, in a wide range of tissues, and in oral and rectal swabs of Egyptian rousettes in this study and in previous experiments [36]. These findings suggest that Egyptian rousettes are generally refractory to ebolavirus infection, implying they are not likely to act as a natural ebolavirus reservoir despite the identification of EBOV-seropositive Egyptian rousettes in Gabon [29] and RESTV-seropositive Asian bats of the *Rousettus amplexicaudatus* and *R. leshenaulti* species [32,33,34]. Furthermore, the Egyptian rousette, which tends to breed well in captivity and can thrive in a laboratory setting, may not be an appropriate experimental model for investigating ebolavirus-reservoir host relationships.

**Figure 8:**
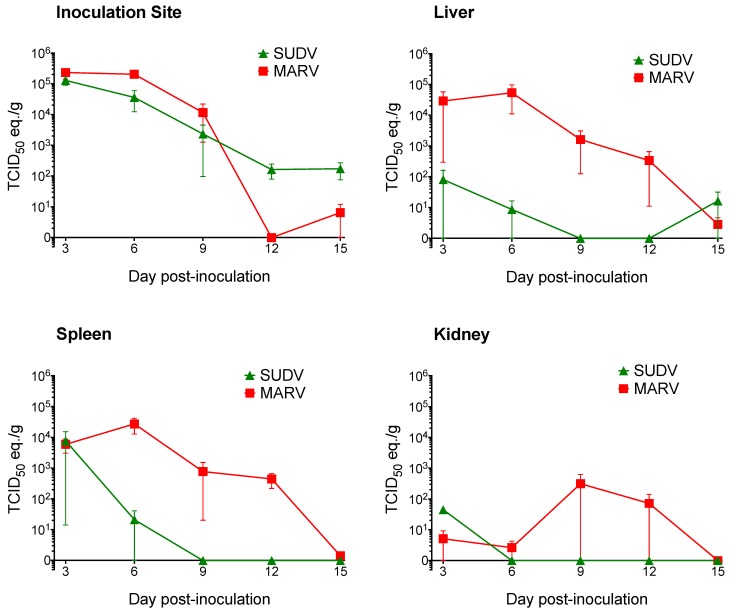
Comparison of Sudan virus and Marburg virus RNA levels in Egyptian rousette tissues (skin at the inoculation site, liver, spleen, and kidney) compared at days 3, 6, 9, 12, and 15 post-infection. Viral loads are expressed as 50% tissue culture infective dose (TCID_50_) equivalents per gram, derived from quantitative reverse-transcriptase PCR. Data for days 3-12 for Marburg virus-inoculated bats are from Amman *et al.* [36].

For Sudan virus, pilot study findings were intermediate: Viral RNA was more widespread than in the other four ebolaviruses, and was detected in both liver and spleen, though animals did not become viremic, viral loads were low, and SUDV antigen was very limited in distribution. These results were replicated and confirmed in a larger serial euthanasia study, which was designed to complement our previous Marburg virus serial euthanasia study [36]; day-by-day comparison of viral RNA levels in key tissues in SUDV and MARV serial euthanasia studies is provided in Figure 8.

SUDV RNA was detected in 10 different tissues, most frequently and at highest loads at 3 and 6 DPI. All 15 bats in the SUDV serial euthanasia study were PCR-positive in at least one tissue between 3 and 15 DPI, but viremia and viral shedding were not identified, and liver and spleen remained IHC-negative. In contrast with Marburg virus, which is frequently found in liver and spleen at levels consistent with replication in these sites, SUDV RNA was only detected in liver or spleen in 3 of 15 bats, and only one bat was PCR-positive in both tissues (Figure 8). The only tissues in which SUDV levels were suggestive of viral replication (TCID_50_/g equivalents greater than inoculation dose) were the inoculation site at days 3 and 6, and the spleen in one bat on day 3. The presence of limited SUDV replication and relatively widespread tissue distribution (though at low levels) indicates that Egyptian rousettes could be more broadly susceptible to infection with Sudan virus than with other ebolaviruses, perhaps given a higher inoculum dose or different route of infection. However, the generally low tissue levels of viral RNA and the lack of any evidence of viral shedding suggest the virus would not be likely to persist in the population. No SUDV outbreak has ever been associated with caves or mines inhabited by Egyptian rousettes. Moreover, SUDV-specific RNA or antibodies have never been identified in any bat species, and the natural reservoir for SUDV remains undiscovered.

Bats inoculated with ebolaviruses did not display any clinical signs or hematologic changes consistent with significant disease, and histologic lesions were minimal. In the pilot ebolavirus study, two individual bats inoculated with either TAFV or RESTV had elevated total white blood cell counts, lymphocytes, and monocytes on two days each. Given that neither bat became viremic and no significant lesions were identified at necropsy, any relationship to viral infection was considered to be unlikely. However, since both bats’ WBC counts had returned to the normal range prior to euthanasia, it is possible that lesions were no longer present at necropsy. CBC values in the SUDV serial euthanasia study remained within normal limits for all bats.

AST was significantly elevated in one BDBV bat at 5 DPI, relative to controls and all other groups, with no associated histologic lesion, CBC abnormality, or significant weight loss. Similarly, in the SUDV serial euthanasia study, AST (but not ALT) was also significantly elevated at 3 DPI relative to controls and to any other day. Increased AST can be caused by liver damage, but also by damage to muscle or erythrocytes, and, in many species AST is less liver-specific than ALT [43]. In other megachiropteran bats, chemical and manual restraint have been shown to be associated with changes in blood chemistry values [44], and restraint-associated myopathy was speculated as a possible cause of increased AST in a survey of wild flying foxes (*Pteropus giganteus*). In this study, it is possible that AST elevation reflects restraint- or capture-associated myopathy rather than leakage from damaged hepatocytes. Creatine kinase (which specifically reflects muscle damage) was not measured, so was not available for correlation.

The Egyptian rousette is a natural host for marburgviruses, and a known source of virus spillover to humans. Unlike marburgviruses, no infectious ebolavirus has ever been isolated from a bat. Evidence supporting a role for bats as reservoir hosts for ebolaviruses is based primarily on ecological and epidemiologic data, which have demonstrated spatiotemporal association and epidemiologic links between human cases of EVD and bats. Though EBOV- and RESTV-seropositive bats have been found in areas where filoviruses have never yet been identified (for example, China [34]), ebolaviral RNA has been detected in bats in only a single study [28]. In our SUDV serial euthanasia study, we showed that two animals developed low SUDV titers without shedding virus, becoming viremic, or supporting widespread viral replication. Thus, though experimental inoculation was sufficient to induce seroconversion, there was no corroborating evidence to support this bat as a likely SUDV reservoir. Similarly, though field serosurveys have identified EBOV-seropositive Egyptian rousettes, these bats were generally refractory to EBOV infection in our pilot study. In contrast, experimental infections of Egyptian rousettes with MARV in this and previous studies [35,36] have replicated many features of natural MARV infections [22,23,25,26], and have expanded our understanding of the virus-reservoir host dynamics.

In conclusion, we have shown that Egyptian rousette bats are not likely to act as sources of ebolavirus spillover in nature. Indeed, the most likely bat candidates for ebolavirus reservoirs are the three species in which both ebolaviral IgG and RNA have been detected (*Epomops franqueti*, *Hypsignathus monstrosus*, and *Myonicteris torquata*) [28]. Our results, in particular the contrasts between ebolaviruses and Marburg virus, suggest the possibility of a one virus-one host species relationship, analogous to that in hantaviruses and rodent species.
